# Heterotopic Kidney Autotransplantation for Recurrent Iatrogenic Distal Ureteral Injury: A Case Report

**DOI:** 10.7759/cureus.38036

**Published:** 2023-04-24

**Authors:** Konstantina Rekouna, Nikolaos Dimitrokallis, Charalampos Kypraios, Dimitrios Kontothanasis, Vasileios Vougas

**Affiliations:** 1 1st Department of Surgery and Transplantation, Evangelismos General Hospital, Athens, GRC; 2 Department of Urology, Evangelismos General Hospital, Athens, GRC

**Keywords:** ureter repair, renal transplant, renal scan, iatrogenic injuries, urinoma, ureteroscopy, pyelogram, autotransplantation, ureter injury

## Abstract

Iatrogenic ureteral injuries are a significant complication during pelvic surgery, requiring a multidisciplinary approach for optimal repair. When a ureteral injury is suspected postoperatively, abdominal imaging is essential to determine the type of injury and thus the timing and method of reconstruction. That can be performed either by a CT pyelogram or by an ureterography-cystography with or without ureter stenting. Although technological advancements and minimally invasive surgery have been gaining ground over open complex surgeries, renal autotransplantation is a well-established technique of proximal ureter repair and should be highly considered when dealing with a severe injury. We hereby report the case of a patient with a recurrent ureter injury and multiple laparotomies treated with autotransplantation, without any major morbidities or change in their quality of life. In every case, a personalized approach for each patient and consultation with experienced transplant experts (surgeons, urologists, and nephrologists) is advised.

## Introduction

Iatrogenic ureteral injuries are a rare complication during pelvic surgery (gynecology, colorectal and vascular surgery, or urology procedures), thus many techniques of reconstruction; surgical or minimally invasive have been described [1]. The method of reconstruction depends on the site of ureteral injury and the time of clinical manifestation. If the injury is presented during surgery primary repair is advised, whereas if the injury presents postoperatively later reconstruction is preferred [2]. Operations involving the area of the pelvis (low anterior resection, hysterectomy, etc.) are associated with distal one-third injuries [2] whereas ureteroscopies cause proximal and middle one-third injuries [3]. In any case, a multidisciplinary approach and collaboration between urologists and pelvic surgeons result in better outcomes. We report the surgical management of a recurrent iatrogenic ureteral injury of a patient with abnormal ureteral anatomy because of several abdominal operations treated with kidney autotransplantation.

## Case presentation

**Figure 1:**
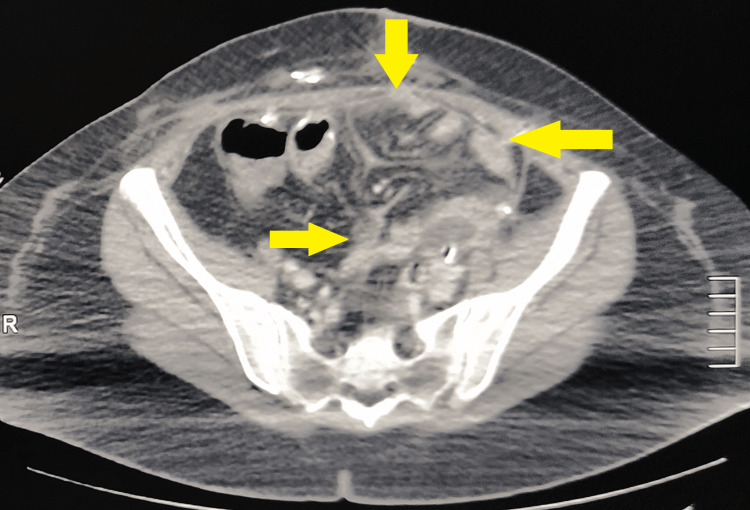
CT urography scan with IV contrast Arrows pointing the fluid collections and urinoma as a result of the ureter injury, as it appeared on the CT scan.

A 48-year-old woman presented in our hospital electively for incisional hernia repair. The patient had a long surgical history including a laparoscopic low anterior resection for a T2N0M0 colorectal cancer. On postoperative day 3, she underwent an emergency laparotomy for per rectum bleeding with the refashioning of the colonic anastomosis and a loop ileostomy. Unfortunately, this was complicated by an iatrogenic injury of the left ureter and therefore a left ureteral repair with ureteroureterostomy was performed. She did not receive any adjuvant therapy and had her stoma reversed six months following her index operation. When presented to our unit five years had passed since her initial diagnosis of colorectal cancer. She had a 25cm incisional hernia taking up the left flank and left lower quadrum. From her oncology follow-up, she remained disease free. She underwent compartment separation surgery, intra-abdominal biological mesh placement, and primary closure of the fascia. After spending a few hours in the recovery area of our hospital she returned to the ward where she became fully ambulatory tolerating oral intake and passing flatus from postoperative day 1. On postoperative day 3, our patient presented with leukocytosis, paralytic ileus, and increased supply in surgical drains. Drain fluid biochemistry reported a creatinine of 37.5 mg/dL with a serum creatinine of 1.2 mg/dL and a urine leak was established. This was investigated further with a computed tomography urography scan with intravenous contrast (Figure 1) combined with a distal ureterography that revealed a complete tear of the left ureter classifying as grade IV injury in the scale of American Association for the Surgery of Trauma (AAST) (Table 1) with localized urinoma and inflammation [4]. The injury had resulted in a 7cm defect. A nephrostomy tube was placed in the left kidney to facilitate urine drainage, absorption of the urinoma, and sterilization of the abdomen. Following this our patient’s clinical condition improved quickly and was finally discharged on day 10.

 

**Table 1:** AAST Classification of ureter injury AAST- American Association for the Surgery of Trauma

Grade	Type of Injury	Description of Injury
I	Hematoma	Contusion or hematoma without devascularization
II	Laceration	<50% transection
III	Laceration	≥50% transection
IV	Laceration	Complete transection with <2cm of devascularization
V	Laceration	Avulsion with >2cm of devascularization

**Figure 2:**
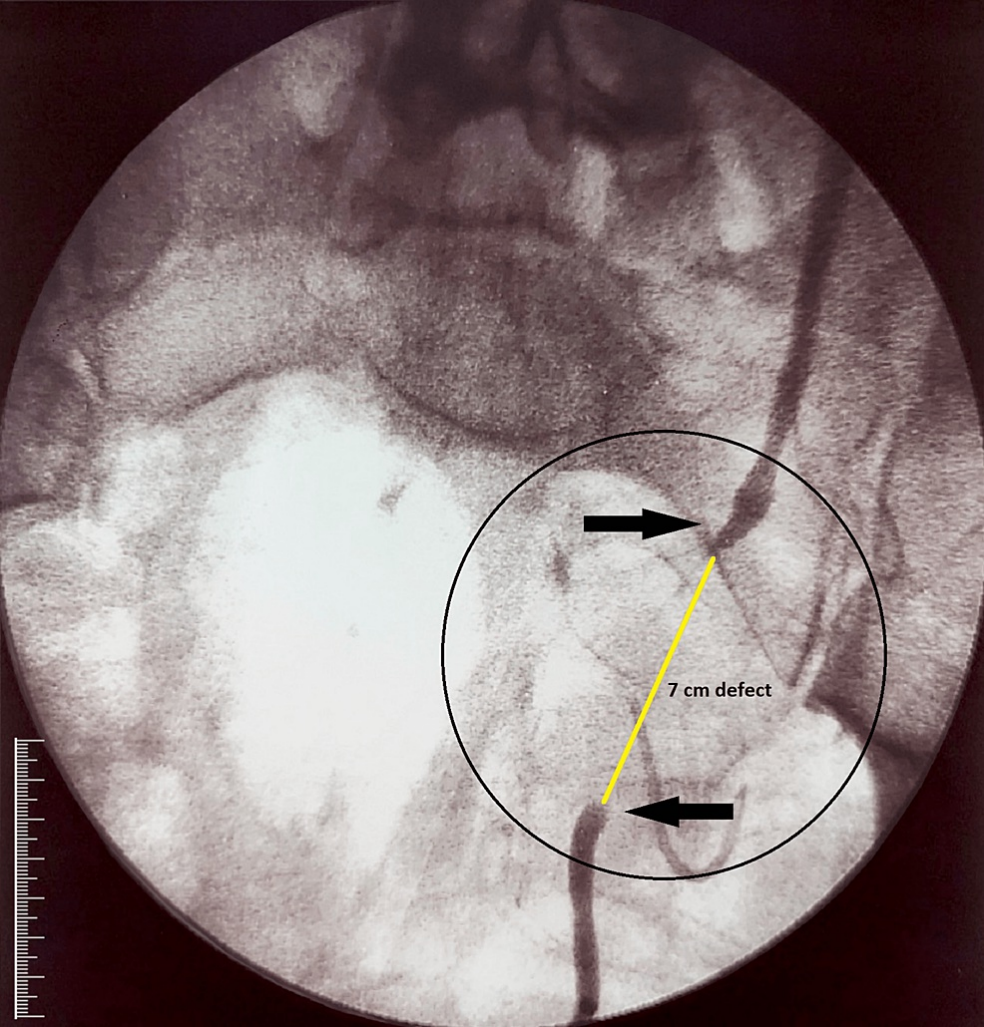
Ureter defect A combined distal and proximal end ureterography was performed, prior to operation, to establish the ureter defect of 7cm. The upper arrow demonstrates the proximal end whereas the lower the distal end of the ureter.

While an outpatient, we went on to carry out repeat ureterography (Figure 2) and computed tomography scans to reassess our patient’s condition and anatomy. Keeping in mind our young patients' age, good performance status with no further comorbidities, the fact that she was cancer-free six years following her initial diagnosis combined with the fact she already had two ureteric injuries, the last one leaving her with a 7cm defect, and five previous laparotomies our multidisciplinary team decided that the best long-term option would be renal autotransplantation. Thus, the patient underwent a left kidney autotransplantation to the right iliac fosa six months following the hernia repair and recurrent ureter injury.

With the patient placed at a left lateral decubitus position, a standard extraperitoneal nephrectomy was carried out through an antero-lateral mini-incision, with careful detachment of the proximal end of the teared ureter to preserve the full length of it (10cm of ureter with good blood flow were preserved, almost one-third of its original length). The kidney was removed, flushed with HTK and placed in cold storage. The surgical wound was closed.

The patient was then shifted to supine position. An oblique incision was performed in the right iliac fossa and the iliac vessels were identified retroperitoneally. The venous and arterial side-to-end anastomoses between the renal and the right iliac vessels were completed by 5-0 polypropelene interrupted sutures. The ureter was reimplanted according to the Lich-Gregoire technique in the bladder with 5-0 polydioxanone interrupted sutures on double J stent [5].

**Figure 3:**
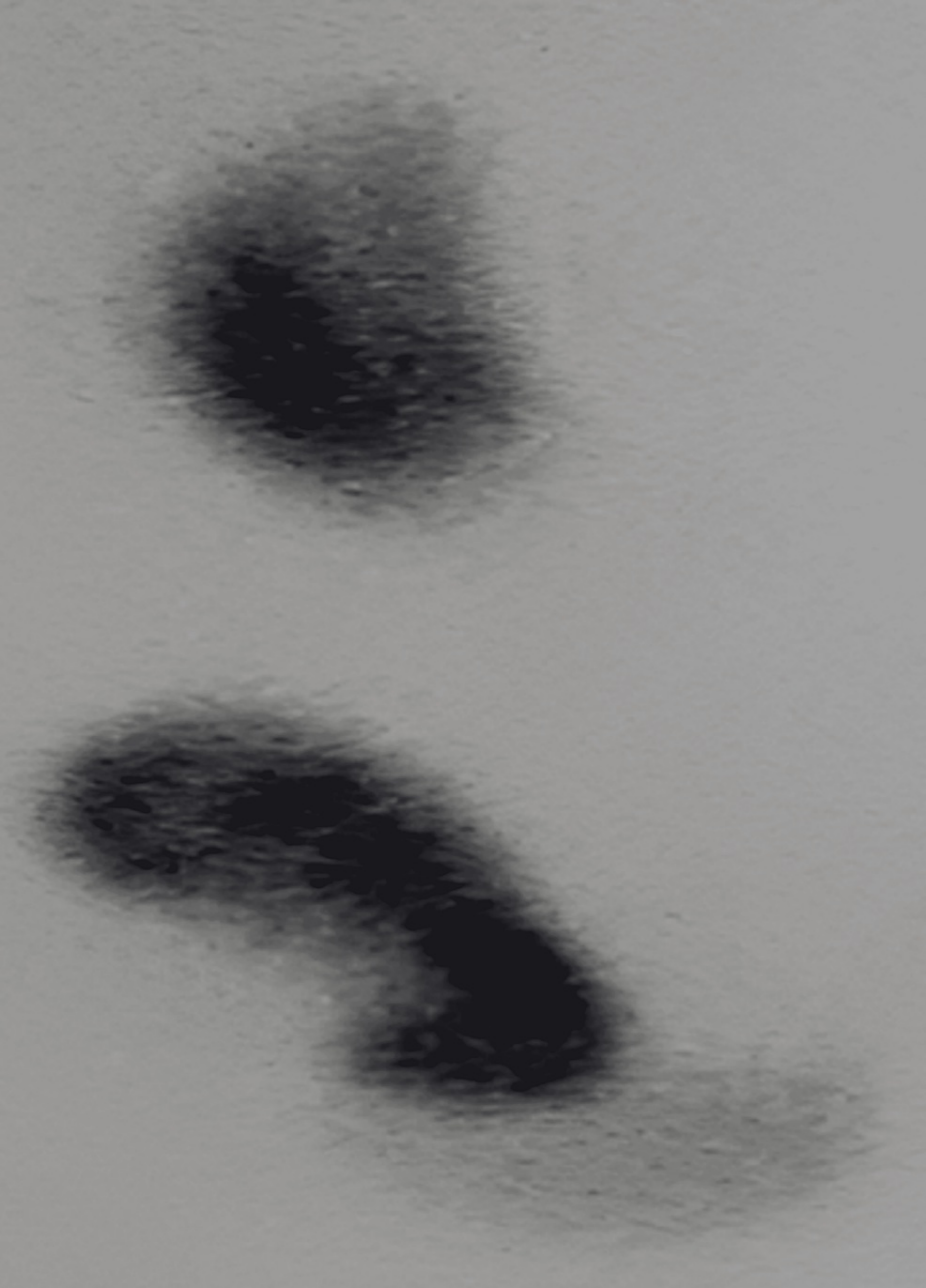
Renal scan Both kidneys on the right side of the patient with normal function as demonstrated by the renal scan (the upper is the native and the lower is the one that was transplanted). The scan was performed one month after the operation.

Overall operating time was 300min for the nephrectomy and 250min for the reimplantantion. Cold ischemia time was 75min. No transfusion was required. The patient was extubated immediately postoperatively and spent two nights in the high dependency unit. Kidney status was checked daily with Doppler ultrasound and laboratory tests. She was ambulatory by postoperative day 2 with normal renal function and no need for dialysis. No significant complication was observed other than low-grade fever due to lung atelectasis that eventually resolved with physiotherapy. She was discharged from the hospital on postoperative day 13. One month after the operation, a renal scan was performed and documented a split function of 58:42 between the two kidneys in favor of the native (Figure 3). The double J stent was removed six weeks postop. The patient has been symptom-free with normal creatinine levels and no evidence of complications at 30 months follow-up.

## Discussion

Iatrogenic ureteral injury remains a challenge during pelvic operations. It is reported as high as 0.25% during rectal procedures [6,7] and varies from 0.02% to 0.78% during gynecology procedures irrespective of the approach being open, laparoscopic, or transvaginal [8,9]. It is also associated with vascular procedures mainly aortofemoral and aortoiliac bypass (0.15%) and last but not least with urologic procedures and ureteroscopies (0.9%-7.4%) mainly for mucosal lesions and urolithiasis [3,9]. The result can be catastrophic, raising the mortality and morbidity of the aforementioned procedures significantly [10]. A surgeon should always strive to distinguish and preserve the ureters during the operation and several technologies and techniques have been introduced toward that end including preoperative ureteric stenting, lighted stents [10], and indocyanine green injection [11], especially during laparoscopic pelvic surgery [12].

Ideally, ureteric injuries should be identified and dealt with intraoperatively however this only happens in 30%-50% of the cases [13]. Unfortunately, we would need to add that in the days of increased use of the electrocautery and ultrasonic shears, ureteric damage can have a late presentation secondary to stricturing caused by thermal injury and mucosa ischemia [3,9].

Should a ureteric injury be suspected, direct visualization through meticulous dissection is advised. Cystoscopy and ureteral catheter placement are other options for ureter interrogation and repair and when a patient position does not allow this could alternatively happen via a cystostomy. Retrograde pyelogram is the most sensitive method to identify ureteral injury and can also serve as a means to treat; however, the fluoroscopic examination is not always possible intraoperatively [9].

Once a patient presents with signs of ureteral injury, cystoscopy, and retrograde pyelogram should be performed to identify the injury and stent if possible. Computed tomography with intravenous pyelogram is another alternative to visualize the relevant anatomy, check for possible injuries and active leaks as well as reveal collections associated with ureteric injuries [4,9].

Distal ureteral injuries are usually treated with ureteroneocystostomy and antireflux ureter reimplantation ideally at the anterior or posterior dome of the bladder to prevent kinking. Should the defect from the ureteral injury be lengthy and tension of the anastomosis is expected, repair with a vesico psoas hitch would be preferred [13,14].

Upper and mid ureteral injuries can be treated with a ureteroureterostomy, especially for small (2-3cm) defects with a running or interrupted anastomosis of the proximal and distal ureter over a stent and covered with peritoneum or other tissue [13].

Should the defects be larger or parts of the ureter not suitable for anastomosis more complex reconstruction, techniques have been proposed such as the Boari tubularized bladder flap where the bladder is incised to create a full-thickness flap that would be mobilized to the proximal ureter end for anastomosis and tubularization. Another complex reconstruction technique would be a transureteroureterostomy where the ureter is mobilized to be anastomosed with the contralateral ureter in an end-to-side fashion. Moreover, several parts of the gastrointestinal tract, mainly the ileum, have been used as a ureteral substitution. However, transureteroureterostomy patients have been reported to require reintervention or revision up to 10% [15] whereas ureteral substitution patients suffer from recurrent infections, strictures, and fistulas [16].

Renal autotransplantation is another well-recognized technique of proximal ureter repair. It was first described by Hardy et al. in 1963 [17]. Since that report, the utility of renal autotransplantation has been proven not only for ureteric injury but also for renal vascular injury and malignancy. In their large retrospective cohort study, Cowan et al. reported 54 consecutive renal autotransplantations. Ureteral stricture and injury were the indications for 20.4% of their patients while other common causes would be chronic renal pain and vascular anomalies. No significant differences in serum creatinine levels were found in a median 21.5 months' follow-up period. Early high-grade complications developed in 14.8% of these patients and these included septic shock and graft thrombosis. The latter were identified in the recovery area and were managed successfully with immediate take back to theatres. Overall in the long-term follow up there was a 3.7% graft loss reported [18].

With the advent of laparoscopic surgery, there has been also a trend toward the laparoscopic harvest of the kidney for renal autotransplantation driving the morbidity of these procedures lower [19]. Several studies have concluded that kidney autotransplantation is a procedure with good long-term results and acceptable complication rates and therefore should be offered where expertise is available [18-20].

## Conclusions

Our patient despite her young age and long-life expectancy had a recurrent iatrogenic injury of the left ureter, resulting in a 7cm defect and uncertainty of the length of the remaining healthy ureter. On top of this, she had a rich surgical history with multiple laparotomies and explorations of the left lower quadrant and the pelvis thus multiple intra-abdominal adhesions and harsh fibrous tissue were to be expected. This case required a permanent non-debilitating solution with less morbidity rates. Thirty months following her autotransplantation, our patient had excellent kidney function and suffered no further complications.

It is our belief that patients with such complex surgical backgrounds and urology complications would benefit from referral to specialized centers where input from surgery, kidney transplant, and urology teams would be available in a multidisciplinary setting. In that way, all available options would be explored and offered. In addition, retrospective data could be collected from these centers and analyzed to provide us with more robust algorithms when consulting such complex cases.
